# Chinese species of the genus ***Aptesis*** Förster (Hymenoptera, Ichneumonidae) parasitizing sawflies, with descriptions of three new species and a key to species

**DOI:** 10.3897/zookeys.290.4781

**Published:** 2013-04-16

**Authors:** Tao Li, Mao-Ling Sheng, Shu-Ping Sun

**Affiliations:** 1 The Key Laboratory for Silviculture and Conservation of Ministry of Education, Beijing Forestry University, Beijing 100083, P. R. China; 2 General Station of Forest Pest Management, State Forestry Administration, 58 Huanghe North Street, Shenyang 110034, P. R. China

**Keywords:** *Aptesis*, Hymenoptera, parasitoid, Cryptinae, Diprionidae, Tenthredinidae, *Neodiprion huizeensis*, *Diprion jingyuanensis*, *Pristiphora erichsonii*, biology

## Abstract

Six species of the genus *Aptesis* Förster 1850 belonging to the tribe Hemigastrini of the subfamily Cryptinae (Hymenoptera, Ichneumonidae) are reported from China. Three of them, *Aptesis elongata* Li & Sheng, **sp. n.**, *Aptesis melana* Li & Sheng, **sp. n.** and *Aptesis nigricoxa* Li & Sheng, **sp. n.** reared from sawflies in China, are new to science. The biology of *Aptesis melana* is described. A key to the species of *Aptesis* known from China is provided.

## Introduction

*Aptesis* Förster 1850, belonging to the subfamily Cryptinae of Ichneumonidae (Hymenoptera), comprises 70 described species, of which five are known from the Oriental region, 16 from the Nearctic, one from the Afrotropical, eight from the Eastern Palaearctic and 42 from the Western Palaearctic (two of them are found across the Palaearctic) (Yu et al. 2012).

Known hosts of *Aptesis* species that have been reliably reared are sawflies of the families Argidae, Diprionidae and Tenthredinidae (Carl 1976; Babendreier 2000; Herz and Heitland 2005; Sawoniewicz 2008; Kasparyan and Kopelke 2009). They parasitize sawfly cocoons (Townes 1970) and are idiobiont ectoparasitoids of larvae and prepupae.

Three species of *Aptesis* Försterhave been known in China: *Aptesis albibasalis* (Uchida, 1930), parasitizing *Arge pagana* (Panzer) (Hymenoptera, Argidae), found in Shandong Province (Wang and Sheng 2006); *Aptesis corniculata* Sheng 2003, parasitizing *Nematus* sp. (Hymenoptera, Tenthredinidae), found in Gansu Province (Sheng and Wu 2003); *Aptesis grandis* Sheng 1998, parasitizing *Diprion jingyuanensis* Xiao & Zhang and *Neodiprion fengningensis* Xiao & Zhou (Hymenoptera, Diprionidae), found in Gansu, Liaoning and Shanxi Provinces (Sheng et al. 1998; Sheng and Chen 2001). In this article, three new species of *Aptesis* parasitizing sawflies are reported.

## Materials and methods

Cocoons of sawflies were collected under the naturally heavily infested trees in Weining County (26°54'N, 104°13'E, elevation 2000 to 2200 m), Guizhou Province (Li et al. 2012). The forest stand is mainly *Pinus armandii* Franch., *Pinus yunnanensis* Franch. and some shrubs.

Pingheliang (33°26'N, 108°28'E, elevation 2000 to 2100 m), Shaanxi Province. The main forest is *Larix principis*-*rupprechtii* Mayr and shrubs. Jialing River (34°13'N, 106°55'E, elevation 1450 to 1600 m), Shaanxi Province. The forest stand is composed of mixed deciduous angiosperms and evergreen conifers, mainly *Larix principis*-*rupprechtii*, *Pinus tabulaeformis* Carr., and some shrubs.

Liupan Mountains (36°10'N, 106°27'E, elevation 1600 to 1800 m), Ningxia Hui Autonomous Region. The forest stand is composed of mixed deciduous angiosperms and evergreen conifers, mainly *Larix principis*-*rupprechtii*, *Pinus tabulaeformis*, *Hippophae rhamnoides* L., *Elaeagnus angustifolia* L. and some shrubs.

Hasi Mountains (37°01'N, 104°22'E, elevation 2200 to 2400 m), Gansu Province. The forest stand is mainly *Pinus tabulaeformis* and shrubs. Maiji Mountains (34°21'N, 105°60'E, elevation 1400 to 1500 m), Gansu Province. The main forest is *Larix principis*-*rupprechtii* and shrubs.

The cocoons were stored individually in glass tubes (100 mm long and 15 mm in diameter) with a piece of filter paper dipped in distilled water in order to prevent desiccation and plugged with absorbent cotton. Cocoons of *Neodiprion huizeensis* Xiao & Zhou (Hymenoptera, Diprionidae) were maintained at 23 ± 1 °C with 60 % to 70 % relative humidity and 14 hours to 10 hours light and dark photoperiod. Cocoons of other sawflies were maintained at room temperature. All cocoons were checked daily for sawflies and parasitoid emergence. Emerged parasitoid larvae and pupae were kept in glass tubes at the same temperature until adult emergence. After emergence of sawflies and parasitoids was complete, all remaining cocoons were dissected and their condition (i.e. status of sawflies, and parasitism) was recorded.

*Aptesis pallidinervis* (Cameron, 1904) was described from India, and there are no host records (Cameron 1904). We were not able to examine specimens of *Aptesis pallidinervis* and we have compared it to our new species based on the original description.

Images of whole insects were taken using a CANON Power Shot A650 IS. Other images were taken using a Cool SNAP 3CCD attached to a Zeiss Discovery V8 Stereomicroscope and captured with QCapture Pro version 5.1. Morphological terminology is based on Gauld (1991). Wing vein nomenclature and terminology based on Mason (1986, 1990).

Type specimens are deposited in the Insect Museum, General Station of Forest Pest Management (GSFPM), State Forestry Administration, P. R. China.

### Terminology

Postocellar line: the shortest distance between the lateral ocelli. Ocular-ocellar line: the shortest distance between the lateral ocellus and the margin of the compound eye. Wing veins referred to in the text are shown on Fig. 9.

## Descriptions

### Genus Aptesis

Förster, 1850

http://species-id.net/wiki/Aptesis

Aptesis Förster 1850. Archiv für Naturgeschichte. 16(1): 71. Type species: *Ichneumon sudeticus* Gravenhorst, 1815. Designated by Viereck 1914.

#### Diagnosis.

Clypeus weakly to rather strongly convex in lateral review, apex truncate or broadly and gently convex, sometimes faintly concave and sometimes with a median tooth.Mandible of moderate length, its lower tooth the same size as upper tooth or sometimes slightly smaller, rarely slightly larger. Tyloids linear to elliptic or subcircular, on about 5 segments, beginning on flagellomeres 9, 10, or 11. Epomia absent or rudimentary. Mesoscutum rather weakly convex. Notaulus vestigial or reaching as much as 0.3 of distance to scutellum. Sternaulus distinct over more than 0.5 of mesopleuron, its end pointing to lower hind corner of mesopleuron. Juxtacoxal carina complete and strong. Propodeum of moderate length to short, with or without weak apophyses. Costula usually present in males, usually incomplete or absent in females, but sometimes absent in males and sometimes complete and strong in females. Propodeal spiracle circular or short elliptic. Areolet pentagonal, moderate size. Median dorsal carinae of first tergum usually weak and reaching about to the spiracle, rarely longer and stronger. Dorsolateral part of postpetiole usually carinate between spiracle and apex, or rarely rounded. Ovipositor sheath about 0.35 times as long as fore wing. Ovipositor straight, tip usually elongate sagittate but sometimes shorter, teeth on lower valve weak, oblique, separated into a basal and an apical series (Townes 1970).

#### Key to species of *Aptesis* known in China

**Table:** 

1	Vein 2rs-m parallel or almost parallel with vein 3rs-m (Figs 31, 34)	2
–	Vein 2rs-m obviously anteriorly convergent with vein 3rs-m (Figs 9, 18)	3
2	Face approximately 2.8 times as wide as long. Mandible teeth of equal length. Postocellar line about 1.3 times as long as ocular-ocellar line. Ovipositor sheath about 0.6 times as long as hind tibia. Metasoma black (Fig. 34)	*Aptesis grandis* Sheng
–	Face approximately 2.0 to 2.1 times as wide as long (Fig. 24). Upper tooth slightly longer than lower tooth. Postocellar line about 1.5 times as long as ocular-ocellar line. Ovipositor sheath about 0.7 times as long as hind tibia. Apical portion of first and second to third terga entirely reddish brown (Fig. 30)	*Aptesis nigricoxa* Li & Sheng, sp. n.
3	Face and frons black (Fig. 11)	4
–	Inner orbits of frons with yellowish white flecks, inner orbits of face with or without yellowish white flecks (Fig. 4)	5
4	Malar space about 1.4 times as long as basal width of mandible. Postocellar line about 1.4 times as long as ocular-ocellar line (Fig. 13). First tergum about 1.3 times as long as apical width. Basal portion of hind tibia dark reddish brown (Fig. 10)	*Aptesis melana* Li & Sheng, sp. n.
–	Malar space about 0.9 times as long as basal width of mandible. Postocellar line about equal with ocular-ocellar line. First tergum about 1.7 times as long as apical width. Basal portion of hind tibia white (Fig. 32)	*Aptesis albibasalis* (Uchida)
5	Face approximately 1.7 times as wide as long (Fig. 2). Vein 2-Cu approximately 2.3 times as long as 2cu-a. Hind wing vein 1-cu about 4.0 times as long as cu-a (Fig. 9). First tergum about 1.5 times as long as apical width	*Aptesis elongata* Li & Sheng, sp. n.
–	Face approximately 2.0 times as wide as long. Vein 2-Cu approximately 1.5 times as long as 2cu-a. Hind wing vein 1-cu about 1.5 times as long as cu-a. First tergum about 1.2 times as long as apical width	*Aptesis corniculata* Sheng

### Aptesis elongata

Li & Sheng sp. n.

urn:lsid:zoobank.org:act:C71C2353-32C7-4DB2-AF95-6538294B184A

http://species-id.net/wiki/Aptesis_elongata

Figures 1−9

#### Etymology.

The name of the new species is based on the elongate area superomedia.

#### Types.

*Holotype*, female, CHINA: Jialing River, Shaanxi Province, 18 May 2010, 2025m, leg. Tao Li. *Paratypes*: 2 females, CHINA: Pingheliang, Shaanxi Province, 29 October 2009, leg. Tao Li; 1 female, CHINA: Pingheliang, Shaanxi Province, 6 April 2010, leg. Tao Li; 1 female, CHINA: Jialing River, Shaanxi Province, 10 May 2010, 2025m, leg. Tao Li. All specimens reared from *Pristiphora erichsonii*, except one female from *Pristiphora xibei* in Pingheliang on 6 April 2010.

#### Diagnosis.

Clypeus about 1.6 times as wide as long. Malar space approximately 1.3 times as long as basal width of mandible. Postocellar line approximately 1.6 times as long as ocular-ocellar line. Antenna with 21 to 23 flagellomeres. Vein 2-Cu approximately 2.3 times as long as 2cu-a. Hind wing vein 1-cu about 4.0 times as long as cu-a. First tergum about 1.5 times as long as apical width. Ovipositor sheath about 0.7 times as long as hind tibia.

#### Description.

**Figures 1–9.**
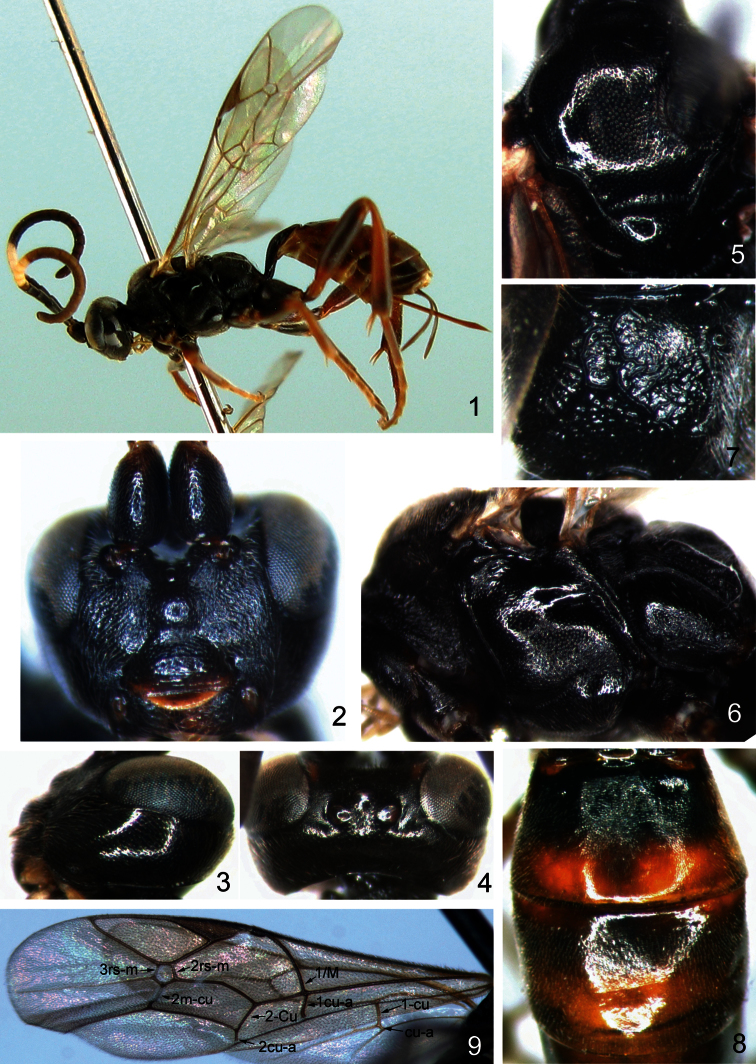
*Aptesis elongata* Li & Sheng, sp. n. Holotype. Female. **1** Body, lateral view **2** Head, anterior view **3** Head, lateral view **4** Head, dorsal view **5** Mesoscutum **6** Mesopleuron **7** Propodeum **8** Terga 2 to 3, dorsal view **9** Fore wing.

Female (Fig. 1). Body length 5.0 to 7.0 mm. Fore wing length 4.0 to 6.0 mm.

**Head.** Face (Fig. 2) about 1.7 times as wide as long, with fine leathery granulose texture and evenly dense punctures; centrally convex; upper margin concave, with a small median protuberance. Clypeus evenly convex, about 1.6 times as wide as long; basally with texture as face, with weak transverse wrinkles; subapex smooth; apical margin flat. Mandible with dense punctures, upper tooth slightly longer than lower tooth. Malar space with fine granulose texture, approximately 1.3 times as long as basal width of mandible. Gena (Fig. 3) smooth with dense fine punctures; median portion weak convex. Ocellar triangle medially with dense fine punctures; remainder of vertex with fine granulose texture (Fig. 4). Postocellar line approximately 1.6 times as long as ocular-ocellar line. Upper portion of frons flat with fine granulose texture; lower portion concave with fine leathery texture. Antenna with 21 to 23 flagellomeres, ratio of length from first to fifth flagellomeres: 7.0:8.0:6.5:5.5:5.0. Occipital carina complete.

**Mesosoma.** Pronotum anteriorly with fine wrinkles and dense punctures; medially with fine oblique wrinkles and fine punctures; upper posterior portion smooth, with fine granulose texture and dense fine punctures. Mesoscutum (Fig. 5) weakly convex, smooth with evenly dense punctures. Notaulus evident approximately on anterior half of mesoscutum. Scutellum evenly convex, smooth with dense fine punctures. Postscutellum transverse, with texture as scutellum. Median portion of mesopleuron (Fig. 6) weakly convex, smooth with evenly fine punctures; lower portion flat, with irregular fine wrinkles and dense fine punctures. Epicnemial carina distinct, approximately reaching subalar ridge; lower portion of subalar ridge weakly concave, with fine transverse wrinkles. Sternaulus distinct, reaching margin of mesopleuron, apically ventral to ventral-posterior corner of mesopleuron. Speculum big, smooth and shiny. Scrobe with strong groove. Metapleuron weakly convex; upper portion smooth with densely fine punctures, lower portion with irregular wrinkles. Juxtacoxal area with fine granulose texture. Juxtacoxal carina and submetapleural carina complete. Legs robust. Ratio of length of hind tarsomeres 1:2:3:4:5 is 19.0:9.0:6.5:4.0:7.0. Fore wing (Fig. 9) with vein 1cu-a opposite or slightly proximal or distal to 1/M. Vein 3rs-m anteriorly convergent with 2rs-m. Vein 2m-cu meets areolet at about mid-point of cell. Vein 2-Cu approximately 2.3 times as long as 2cu-a. Hind wing vein 1-cu about 4.0 times as long as cu-a. Propodeum (Fig. 7) weakly convex. Area basalis an inverse trapezium, small. Area superomedia elongate, approximately 1.3 times as long as wide, with distinct transverse wrinkles, central portion weakly concave. Costula connecting area superomedia at its middle. Posterior transverse carina of propodeum strong. Area petiolaris weakly sloping, with irregular transverse wrinkles. Area externa with fine granulose texture. Area dentipara with irregular oblique wrinkles. Propodeal spiracle circular, moderately large, near pleural carina, located at anterior 0.2 of propodeum.

**Metasoma.** First tergum about 1.5 times as long as apical width, with fine leathery texture, smooth. Median dorsal carinae distinct, extending beyond spiracle. Dorsolateral and ventrolateral carinae complete. Spiracle circular, very small, located at apical 0.3 times of first tergum. Second tergum (Fig. 8) approximately 0.7 times as long as apical width, with fine granulose texture; centrally with weak wrinkles. Thyridia present. Third tergum about 0.6 times as long as basal width, with texture as first tergum; basally polished. Fourth to eighth terga with texture as third tergum. Ovipositor sheath about 0.7 times as long as hind tibia. Ovipositor straight and slender, apical portion sharp, with nodus. Apical portion of lower valve with weak ridges.

**Color** (Fig. 1)**.** Black, except the following. Flecks of inner orbits on frons yellowish brown with some reddish. Maxillary and labial palpi, apical half of fifth and sixth to ninth flagellomeres, tegula laterally, wing base, yellow. Fore leg (coxa, trochanters, most of femur, blackish brown), mid leg (coxa, trochanters, most of femur, blackish brown), hind leg (coxa, first trochanter, most of femur, apical portion of tibia, blackish brown), yellowish brown with some reddish. Apical half of second tergum, thyridia, third tergum (medially dark reddish brown), base of fourth tergum, reddish brown. Wing membrane brownish hyaline. Veins, pterostigma, blackish brown.

**Male.** Unknown.

#### Hosts.

*Pristiphora erichsonii*, *Pristiphora xibei* (Hymenoptera: Tenthredinidae).

#### Host plant.

*Larix principis*-*rupprechtii* (Pinaceae).

#### Biology.

The mature larva forms a cocoon inside the host’s cocoon and outside the body of the host larva.

#### Remarks.

This new species is similar to *Aptesis corniculata* Sheng 2003, but can be distinguished from the latter by the following combination of characters: face approximately 1.7 times as wide as long; fore wing vein 2-Cu approximately 2.3 times as long as 2cu-a; hind wing vein 1-cu about 4.0 times as long as cu-a; first tergum about 1.5 times as long as apical width. *Aptesis corniculata*: face approximately 2.0 times as wide as long; fore wing vein 2-Cu approximately 1.5 times as long as 2cu-a; hind wing vein 1-cu about 1.5 times as long as cu-a; first tergum about 1.2 times as long as apical width.

### Aptesis melana

Li & Sheng sp. n.

urn:lsid:zoobank.org:act:D0C50573-9CFC-42F4-BAA6-A81B2BF11705

http://species-id.net/wiki/Aptesis_melana

Figures 10−18

#### Etymology.

The name of the new species is based on the black body color.

#### Types.

*Holotype*, female, CHINA: Weining County, Guizhou Province, 20 February 2012, leg. Mao-Ling Sheng. *Paratypes*: 2 females, CHINA: Jialing River, Shaanxi Province, 8 May 2010, 2025m, leg. Tao Li; 2 females, CHINA: Pingheliang, Shaanxi Province, 16 to 24 May 2010, leg. Tao Li; 6 females, CHINA: Maiji Mountains, Gansu Province, 8 to 24 May 2010, leg. Tao Li; 35 females and 15 males, CHINA: Liupan Mountains, Ningxia Hui Autonomous Region, 8 May to 1 August 2011, leg. Tao Li; 337 females and 199 males, CHINA: Weining County, Guizhou Province, 17 February to 3 April 2012, leg. Tao Li, Mao-Ling Sheng; 11 females and 5 males, CHINA: Liupan Mountains, Ningxia Hui Autonomous Region, 17 to 23 May 2012, leg. Tao Li. All specimens reared from *Pristiphora erichsonii* in Jialingjiang River, Pingheliang, Maiji Mountains and Liupan Mountains, except one reared from *Neodiprion huizeensis* in Weining County, Guizhou Province.

#### Diagnosis.

Clypeus approximately 1.7 times as wide as long. Mandible teeth about equal in length. Malar space approximately 1.4 times as long as basal width of mandible. Postocellar line approximately 1.4 times as long as ocular-ocellar line. Antenna with 25 flagellomeres. Fore wing with vein 1cu-a opposite 1/M; Vein 2-Cu approximately 1.5 times as long as 2cu-a. First tergum about 1.3 times as long as apical width. Ovipositor sheath about 0.8 times as long as hind tibia.

#### Description.

**Figures 10–18.**
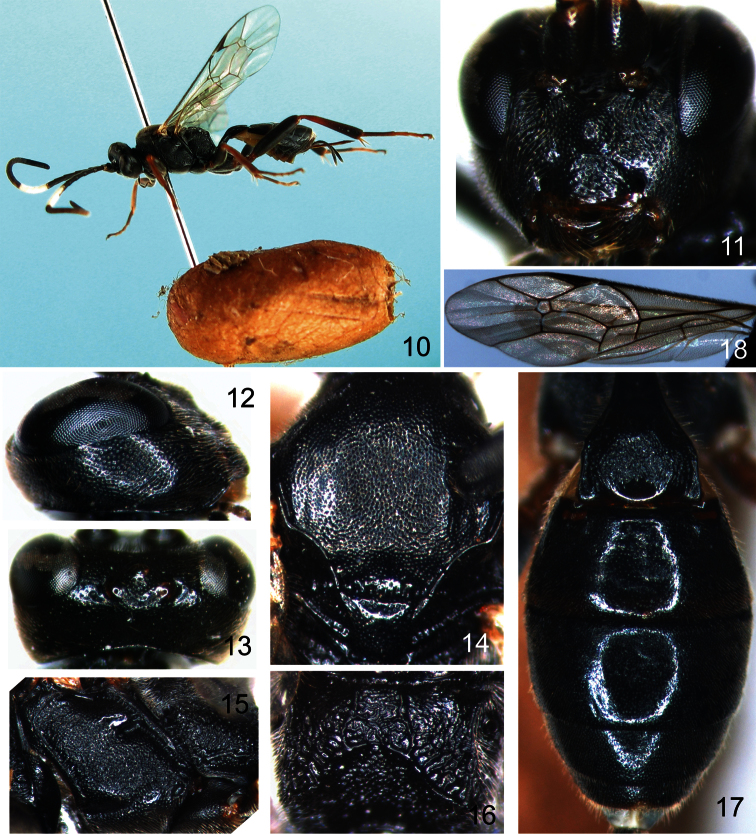
*Aptesis melana* Li & Sheng, sp. n. Holotype. Female. **10** Body, lateral view **11** Head, anterior view **12** Head, lateral view **13** Head, dorsal view **14** Mesoscutum **15** Mesopleuron **16** Propodeum **17** Metasoma, dorsal view **18** Fore wing.

Female (Fig. 10). Body length 6.0 to 9.0 mm. Fore wing length 5.0 to 7.0 mm.

**Head.** Face (Fig. 11) about 2.7 times as wide as long, with dense fine punctures; centrally convex, punctures smaller than on remainder of face; upper margin concave. Clypeus approximately 1.7 times as wide as long; basally with dense fine punctures; apically with weak wrinkles; apical margin flat. Mandible with dense punctures, teeth approximately equal in length. Malar space with fine leathery texture, approximately 1.4 times as long as basal width of mandible. Gena (Fig. 12) with evenly dense punctures. Vertex (Fig. 13) with texture as gena. Outside of ocellar triangle with sparse punctures. Postocellar line approximately 1.4 times as long as ocular-ocellar line. Frons with evenly dense punctures, basally smooth. Antenna with 25 flagellomeres, ratio of length from first to fifth flagellomeres: 12.0:11.0:10.0:9.0:7.0. Occipital carina complete.

**Mesosoma.** Pronotum anteriorly with weak wrinkles; medially with short transverse wrinkles; with dense punctures dorso-posteriorly. Mesoscutum (Fig. 14) flat, with dense punctures. Notaulus present anteriorly. Scutellum with punctures sparser than on mesoscutum. Postscutellum transverse, smooth. Mesopleuron (Fig. 15) with texture as mesoscutum, with irregular wrinkles. Epicnemial carina strong, reaching subalar ridge. Sternaulus distinct, reaching hind margin of mesopleuron, apically ventral to ventral-posterior corner of mesopleuron. Scrobe with strong groove. Metapleuron with texture as mesopleuron. Juxtacoxal carina incomplete. Submetapleural carina complete. Legs robust. Ratio of length of hind tarsomeres 1:2:3:4:5 is 13.0:5.0:4.0:2.0:4.0. Fore wing (Fig. 18) with vein 1cu-a opposite 1/M. Vein 3rs-m anteriorly convergent with 2rs-m. Areolet receiving vein 2m-cu approximately at its middle. Vein 2-Cu approximately 1.5 times as long as 2cu-a. Hind wing vein 1-cu about 4.0 times as long as cu-a. Propodeum (Fig. 16) weakly convex. Anterior transverse carina absent. Areas basalis and superomedia combined, with irregular wrinkles. Areas externa and dentipara combined; basal portion with sparse punctures and weak wrinkles; apically with irregular short wrinkles. Posterior transverse carina strong. Area petiolaris sloping, with irregular wrinkles; medially weakly concave. Propodeal apophysis distinct. Propodeal spiracle approximately circular, located at anterior 0.2 of propodeum.

**Metasoma** (Fig. 17)**.** First tergum about 1.3 times as long as apical width, apically with weak wrinkles. Median dorsal carinae distinct, basally parallel. Dorsolateral and ventrolateral carinae complete. Spiracle circular, small, located at apical 0.3 of first tergum. Second tergum approximately 0.5 times as long as apical width, smooth. Third to eighth terga with texture as second tergum. Ovipositor sheath about 0.8 times as long as hind tibia. Ovipositor strong, straight, apically sharp. Apical portion of lower valve with 2 ridges.

**Color** (Fig. 10)**.** Black, except the following. Sixth to ninth (base of tenth) flagellomeres, wing base, yellowish white. Apical portion of clypeus, mandible (basally and teeth, blackish brown), fore leg (part of coxa black; first trochanter, femur laterally, blackish brown), mid leg (coxa, femur, blackish brown), hind leg (coxa, femur, tibia apically, first tarsomere, blackish brown), reddish brown. Maxillary and labial palpi, pterostigma, veins, blackish brown. Wing membrane brownish hyaline.

**Male.** Body length 7.0 to 8.0 mm. Fore wing length 5.0 to 7.0 mm. Body black. Face, clypeus, mandible (teeth black), maxillary and labial palpi, wing base, yellowish white. Fore leg (most of coxa, trochanters, second to forth tarsomeres, yellowish white; fifth tarsomere brown), mid leg (part of coxa, trochanters, second to fourth tarsomeres, yellowish white; most of femur blackish brown; most of first and fifth tarsomeres, brown), hind leg (coxa, trochanters, femur, most of tibia, blackish brown; first and fifth tarsomeres basally brown; Apex of first and second to fourth tarsomeres, yellowish white), reddish brown.

#### Hosts.

*Neodiprion huizeensis* (Hymenoptera: Diprionidae), *Pristiphora erichsonii* (Hymenoptera: Tenthredinidae).

#### Host plants.

*Pinus armandi*, *Larix principis*-*rupprechtii* (Pinaceae).

#### Biology.

**Figures 19–22.**
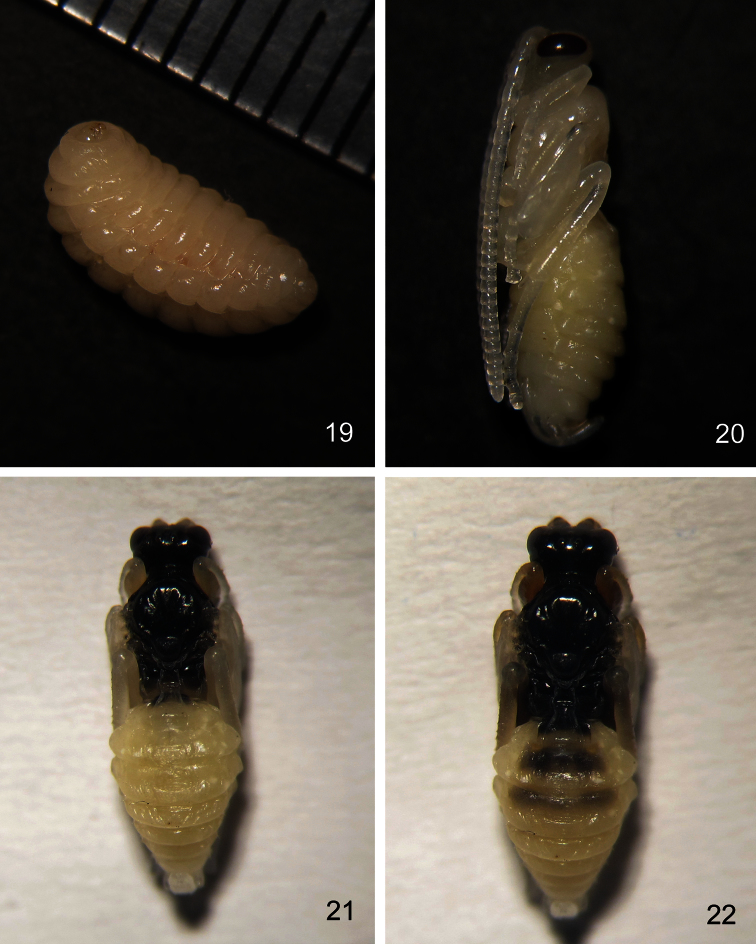
*Aptesis melana* Li & Sheng, sp. n. **19** Larva. **20, 21, 22** Pupa.

The mature larva of *Aptesis melana* forms a cocoon outside the body of the *Neodiprion huizeensis* larva and inside the cocoon of the host. The mature larva of *Aptesis melana* is cream-colored (Fig. 19). The pupa changes continuously as development continues. The young pupa is cream-colored, the compound eyes light red. After three days, the compound eyes change to dark red (Fig. 20). Two days later, the head and mesosoma are blackish brown (Fig. 21). After two days, the head and mesosoma change to black; the first and second terga blackish brown, third to eighth terga brown (Fig. 22); the basal portion of flagellomeres brown, median portion yellowish white and apical portion yellowish brown. The mature pupa is the same color as the adult. Of 537 *Aptesis melana* reared from *Neodiprion huizeensis*, the female to male ratio was 1.7: 1. The average parasitism rate of *Neodiprion huizeensis* by *Aptesis melana* was 8.7%. Adults of *Aptesis melana* emerged between 17th February to 12th March under laboratory conditions.

#### Remarks.

This new species is similar to *Aptesis albibasalis* (Uchida, 1930) but can be distinguished from the latter by the following combination of characters: malar space about 1.4 times as long as basal width of mandible; postocellar line about 1.4 times as long as ocular-ocellar line; first tergum about 1.3 times as long as apical width; hind tibia basally dark reddish brown. *Aptesis albibasalis*: malar space about 0.9 times as long as basal width of mandible; postocellar line about equal with ocular-ocellar line; first tergum about 1.7 times as long as apical width; hind tibia basally white.

### Aptesis nigricoxa

Li & Sheng sp. n.

urn:lsid:zoobank.org:act:89FF2D20-D0A2-41B4-AC95-64CA314F48B2

http://species-id.net/wiki/Aptesis_nigricoxa

Figures 23−31

#### Etymology.

The name of the new species is based on the black coxae.

#### Types.

*Holotype*, female, CHINA: Weining County, Guizhou Province, 26 February 2012, leg. Mao-Ling Sheng. *Paratype*: 1 female, CHINA: Weining County, Guizhou Province, 1 March 2012, leg. Tao Li. Both specimens reared from *Neodiprion huizeensis*.

#### Diagnosis.

Clypeus about 1.9 to 2.0 times as wide as long. Malar space approximately 1.2 to 1.4 times as long as basal width of mandible. Postocellar line approximately 1.5 times as long as ocular-ocellar line. Antenna with 30 to 31 flagellomeres. Fore wing vein 3rs-m almost parallel with 2rs-m. Vein 2-Cu approximately 1.5 times as long as 2cu-a. First tergum about 1.5 times as long as apical width. Ovipositor sheath about 0.7 times as long as hind tibia.

#### Description.

**Figures 23–31.**
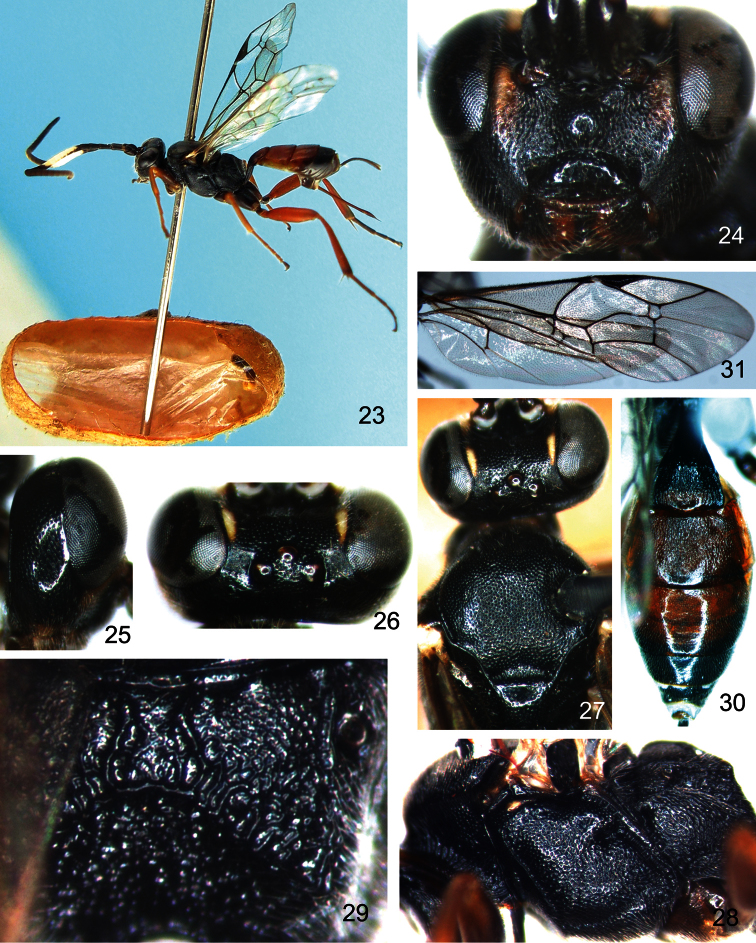
*Aptesis nigricoxa* Li & Sheng, sp. n. Holotype. Female. **23** Body, lateral view **24** Head, anterior view **25** Head, lateral view **26** Head, dorsal view **27** Mesoscutum **28** Mesopleuron **29** Propodeum **30** Metasoma, dorsal view **31** Fore wing.

Female (Fig. 23). Body length 6.0 to 8.5 mm. Fore wing length 6.0 to 7.0 mm.

**Head.** Face (Fig. 24) about 2.0 to 2.1 times as wide as long, with short brown setae and dense punctures; centrally weakly convex, punctures sparser than remainder of face; face orbits granulose with sparse punctures. Epistomal suture distinct. Clypeus about 1.9 to 2.0 times as wide as long, with sparse punctures; basally evenly convex; apically flat, with transverse wrinkles, apical margin distinct. Mandible strong, with dense fine punctures; upper tooth slightly longer than lower tooth. Malar space with fine granulose texture, approximately 1.2 to 1.4 times as long as basal width of mandible. Gena (Fig. 25) smooth, with dense fine punctures; centrally weakly convex. Ocellar triangle densely punctate; outside of ocellar triangle with fine leathery texture and sparse fine punctures (Fig. 26). Postocellar line about 1.5 times as long as ocular-ocellar line. Frons densely punctate, medially weak transverse convexity, evenly concave ventrally, smooth with weak wrinkles; orbits of frons with sparse punctures. Antenna with 30 to 31 flagellomeres, ratio of length from first to fifth flagellomeres: 7.0:8.0:7.0:6.0:5.0. Occipital carina complete.

**Mesosoma.** Pronotum anteriorly with weak wrinkles and fine punctures; medially with weak transverse wrinkles; with dense punctures dorso-posteriorly. Mesoscutum (Fig. 27) weakly convex, with texture as upper posterior of pronotum, punctures relatively coarse. Notaulus distinct. Punctures of scutellum sparser than on mesoscutum. Postscutellum transverse, with sparse fine punctures. Mesopleuron (Fig. 28) flat, with texture as mesoscutum. Epicnemial carina distinct, reaching subalar ridge. Sternaulus reaching hind margin of mesopleuron, apically ventral to ventral-posterior corner of mesopleuron. Speculum small. Scrobe with strong groove. Metapleuron with texture as mesopleuron, ventrally with irregular wrinkles. Juxtacoxal carina distinct. Leg robust. Ratio of length of hind tarsomeres 1:2:3:4:5 is 21.0:10.0:6.0:4.0:6.0. Fore wing (Fig. 31) with vein 1cu-a opposite 1/M. Vein 3rs-m almost parallel with 2rs-m. Areolet receiving vein 2m-cu approximately at its middle. Vein 2-Cu approximately 1.5 times as long as 2cu-a. Hind wing vein 1-cu about 4.0 times as long as cu-a. Propodeum (Fig. 29) with irregular wrinkles. Anterior transverse carina absent. Posterior transverse carina strong. Areas basalis and superomedia combined, with irregular wrinkles. Area petiolaris sloping. Propodeal spiracle almost circular, located at anterior 0.2 of propodeum.

**Metasoma** (Fig. 30)**.** First tergum about 1.5 times as long as apical width, smooth with weak wrinkles. Median dorsal carinae distinct, almost parallel. Dorsolateral and ventrolateral carinae complete. Spiracle circular, small, located at apical 0.3 of first tergum. Second tergum about 0.6 times as long as apical width, mostly with fine longitudinal wrinkles, apically smooth with fine punctures. Third to eighth terga shiny, densely punctate. Ovipositor sheath about 0.7 times as long as hind tibia. Ovipositor straight, strong, with small nodus, apically sharp.

**Color** (Fig. 23)**.** Black, except the following. Face orbits, flecks of inner orbits on frons, wing base, fifth flagellomere apically and sixth to tenth, eleventh basally, yellowish white. Mandible (basally and teeth, blackish brown), apical margin of first tergum, second to third terga, fourth tergum (apically blackish brown), reddish brown. Fore leg (coxa blackish brown with reddish brown, trochanters blackish brown), mid leg (coxa, trochanters, blackish brown), hind leg (coxa, first trochanter, tibia apically, tarsomeres, blackish brown), red. Wing membrane brownish hyaline. Pterostigma and veins blackish brown.

**Male.** Unknown.

#### Host.

*Neodiprion huizeensis* (Hymenoptera: Diprionidae).

#### Host plant.

*Pinus armandi* (Pinaceae).

#### Biology.

Adults of *Aptesis nigricoxa* emerged from overwintering cocoons of *Neodiprion huizeensis*.

#### Remarks.

Based on the original description, this new species is similar to *Aptesis pallidinervis* (Cameron, 1904) but can be distinguished from the latter by the following combination of characters: apical portion of fifth and sixth to tenth and basal portion of eleventh flagellomeres, yellowish white; mandible teeth blackish brown; pterostigma blackish brown; metasoma black; postpetiole with weak wrinkles. *Aptesis pallidinervis*: eighth to thirteenth flagellomeres white; mandible teeth rufo-testaceous; pterostigma pale yellow; apex of first, second to third terga ferruginous, seventh to eighth terga white; Base of postpetiole with strong longitudinal wrinkles.

### Aptesis albibasalis

(Uchida, 1930)

http://species-id.net/wiki/Aptesis_albibasalis

Figure 32

Plectocryptus albibasalis Uchida, 1930. Journal of the Faculty of Agriculture, Hokkaido Imperial University, 25(4): 327.

#### Specimens examined.

1 female, CHINA: Jinan, Shandong Province, 7 October 2004, leg. Mao-Ling Sheng. 10 females and 4 males, CHINA: Jinan, Shandong Province, 21 to 26 December 2004, leg. Nan-Xi Wang, Mao-Ling Sheng. 6 females, CHINA: Zhongmu County, Henan Province, 4 to 14 April 2011, leg. Tao Li. All specimens reared from *Arge pagana* (Panzer).

**Host.** *Arge pagana* (Hymenoptera: Argidae).

**Host plant.** *Rosa chinensis* Jacq. (Rosaceae).

**Distribution.** China (Henan, Shandong), Korea, Japan, Russia (Wang and Sheng 2006; Yu et al. 2012).

**Figure 32.**
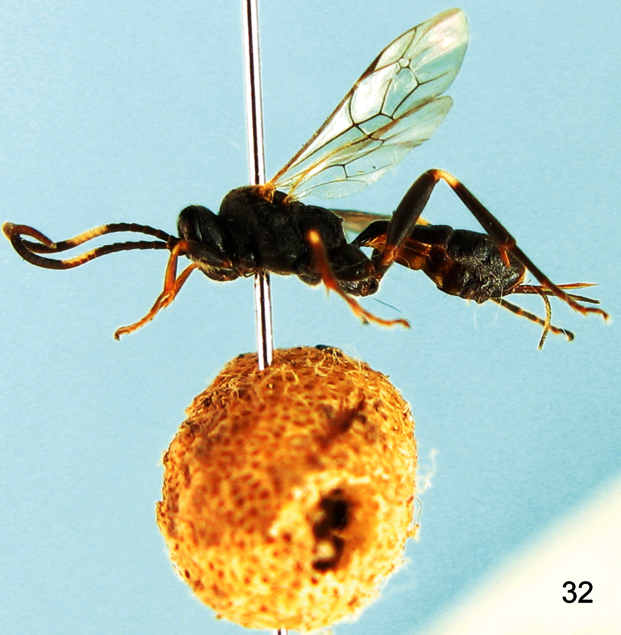
*Aptesis albibasalis* (Uchida, 1930). Female. Body, lateral view.

### Aptesis corniculata

Sheng, 2003

http://species-id.net/wiki/Aptesis_corniculata

Figure 33

Aptesis corniculata Sheng, 2003. Entomotaxonomia, 25(2): 148.

#### Specimens examined.

3 females and 1 male, CHINA: Tianshui, Gansu Province, 26 March to 4 April 2001, leg. Xing-Yu Wu. All specimens reared from *Nematus* sp. (Sheng and Wu 2003).

#### Host.

*Nematus* sp. (Hymenoptera: Tenthredinidae).

#### Host plant.

*Salix* sp. (Salicaceae).

#### Distribution.

China (Gansu).

**Figure 33.**
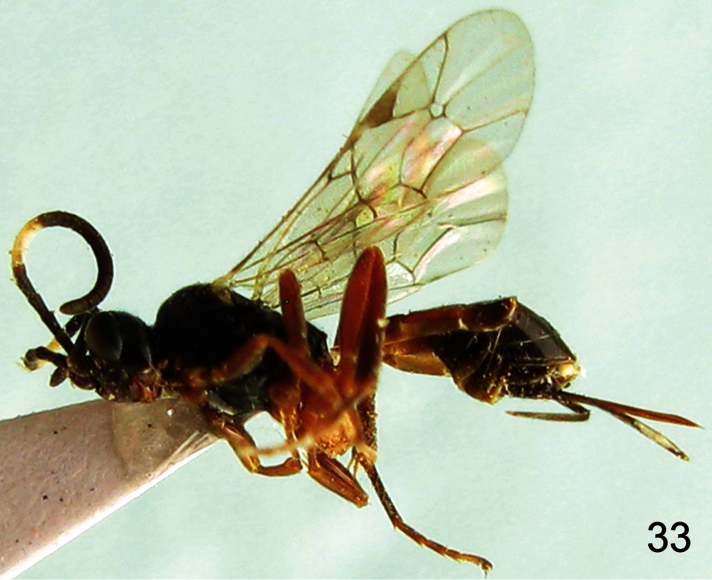
*Aptesis corniculata* Sheng, 2003. Female. Body, lateral view.

### Aptesis grandis

Sheng, 1998

http://species-id.net/wiki/Aptesis_grandis

Figure 34

Aptesis grandis Sheng, 1998. Acta Entomologica Sinica, 41(3): 316.

#### Specimens examined.

1 female and 2 males, CHINA: Qinyuan, Shanxi Province, 13 June 1994, leg. Mao-Ling Sheng. 11 females and 38 males, CHINA: Qinyuan, Shanxi Province, 20 June 1995, leg. Guo-Fa Chen, Qing-He Zhang, reared from *Diprion jingyuanensis* (Sheng and Chen 2001). 6 females and 2 males, CHINA: Hasi Mountains, Gansu Province, 4 to 30 June 2010, leg. Tao Li, reared from *Diprion jingyuanensis*. 130 females and 101 males, CHINA: Weining County, Guizhou Province, 19 February to 30 March 2012, leg. Tao Li, Mao-Ling Sheng, reared from *Neodiprion huizeensis*.

#### Hosts.

*Diprion jingyuanensis*, *Neodiprion fengningensis* (Sheng and Chen 2001), *Neodiprion huizeensis* (Hymenoptera: Diprionidae).

#### Host plants.

*Pinus tabulaeformis*, *Pinus armandi*, *Picea crassifolia* Kom. (Pinaceae).

#### Distribution.

China (Gansu, Guizhou, Liaoning, Shanxi) (Yu et al. 2012).

#### Biology.

The mature larva of *Aptesis grandis* forms a cocoon outside the body of the *Neodiprion huizeensis* larva and inside the cocoon of the host. Of 231 adults of *Aptesis grandis* that emerged from cocoons of *Neodiprion huizeensis*, the female to male ratio was 1.2: 1. The average parasitism rate of *Neodiprion huizeensis* by *Aptesis grandis* was 3.8%. Adults of *Aptesis grandis* emerged between 20th February to 9th March under laboratory conditions.

**Figure 34.**
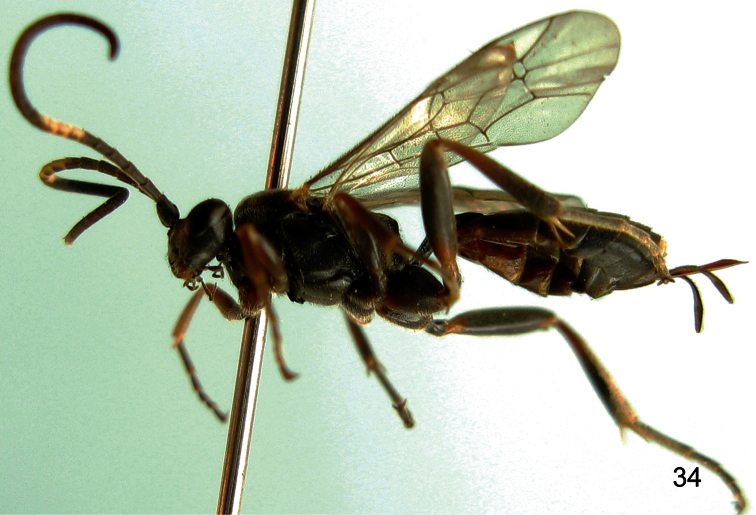
*Aptesis grandis* Sheng, 1998. Female. Body, lateral view.

## Supplementary Material

XML Treatment for Aptesis

XML Treatment for Aptesis elongata

XML Treatment for Aptesis melana

XML Treatment for Aptesis nigricoxa

XML Treatment for Aptesis albibasalis

XML Treatment for Aptesis corniculata

XML Treatment for Aptesis grandis
